# Bowel monitoring in psychiatry of old age: a quality improvement project

**DOI:** 10.1192/bjo.2021.489

**Published:** 2021-06-18

**Authors:** Alistair Cowie, Stephanie Sloan, Cara McDonald

**Affiliations:** NHS Tayside

## Abstract

**Aims:**

This project aims to ensure all patients in the dementia ward 1 in Kingsway Care Centre, Dundee have daily bowel monitoring and achieve a normal bowel habit. The hypothesis is that patients are inadequately screened and substantial undiagnosed constipation exists.

**Background:**

Constipation has a prevalence of 16-50% among individuals over 65 years old in the community. Psychiatric illnesses are known risk factors with older psychiatric patients 3-6 times more likely to be constipated. Untreated constipation may progress to serious complications such as bowel obstruction and bowel perforation. Delirium, often mislabelled as worsening psychiatric symptoms, also may occur leading to additional psychotropic medications being prescribed, further worsening the constipation.

**Method:**

All patients in Ward 1, Kingsway Care Centre Dundee over 4 months were included, amounting to 25 patients. Data were gathered from stool charts weekly. Quality improvement framework was followed with two plan-do-study-act (PDSA) cycles completed. Normal bowel function was assessed against ROME IV constipation criteria and less than 75% of Bristol stool type 6 or 7 due to the risk of overflow diarrhoea and laxative overuse. In the first PDSA cycle, stool charts were modified to account for patients independently mobilising to the bathroom and daily documentation even if bowel movements were uncertain. The second PDSA cycle introduced a sticker on charts folder to “ask the patient” along with a staff education leaflet on the complications of constipation. Data were anonymised and analysed with run charts using Microsoft Excel.

**Result:**

At baselines, 50% of patients had a stool chart. This increased to 90% in cycle 1, 100% in cycle 2. 28% of patients had any stools documented at baselines. This increased to 31% in cycle 1, 59% in cycle 2. At baselines, 0% of patients had a normal bowel habit. This maintained at 0% in cycle 1 but increased to 13% in cycle 2. No serious complications were found in patients assisted with toileting. However, 34% of independently mobile patients developed serious complications.

**Conclusion:**

Poor documentation existed in all patients, particularly those independently mobile. Independently mobile patients were particularly at risk of serious complications of constipation compared to assisted patients. Introduction of new stool charts in the first PDSA cycle resulted in increased documentation but limited benefit for identification of constipation. The second PDSA cycle, targeting staff education and compliance, showed an increase in identification of constipation indicating limited staff knowledge as a key barrier to improvement in patients’ bowel habit.

